# BCR-ABL Independent Mechanisms of Resistance in Chronic Myeloid Leukemia

**DOI:** 10.3389/fonc.2019.00939

**Published:** 2019-09-24

**Authors:** Federica Loscocco, Giuseppe Visani, Sara Galimberti, Antonio Curti, Alessandro Isidori

**Affiliations:** ^1^Haematology and Haematopoietic Stem Cell Transplant Center, AORMN Hospital, Pesaro, Italy; ^2^Department of Clinical and Experimental Medicine, University of Pisa, Pisa, Italy; ^3^Department of Experimental, Diagnostic and Specialty Medicine, Institute of Hematology L. and A. Seràgnoli, University of Bologna, Bologna, Italy

**Keywords:** chronic myeloid leukemia, leukemia stem cells, TKI, resistance, BCR-ABL-independent mechanisms, microenvironment, epigenetic, immune system

## Abstract

Not all chronic myeloid leukemia (CML) patients are cured with tyrosine kinase inhibitors (TKIs), and a proportion of them develop resistance. Recently, continuous BCR-ABL gene expression has been found in resistant cells with undetectable BCR-ABL protein expression, indicating that resistance may occur through kinase independent mechanisms, mainly due to the persistence of leukemia stem cells (LSCs). LSCs reside in the bone marrow niche in a quiescent state, and are characterized by a high heterogeneity in genetic, epigenetic, and transcriptional mechanisms. New approaches based on single cell genomics have offered the opportunity to identify distinct subpopulations of LSCs at diagnosis and during treatment. In the one hand, TKIs are not able to efficiently kill CML-LSCs, but they may be responsible for the modification of some LSCs characteristics, thus contributing to heterogeneity within the tumor. In the other hand, the bone marrow *niche* is responsible for the interactions between surrounding stromal cells and LSCs, resulting in the generation of specific signals which could favor LSCs cell cycle arrest and allow them to persist during treatment with TKIs. Additionally, LSCs may themselves alter the niche by expressing various costimulatory molecules and secreting suppressive cytokines, able to target metabolic pathways, create an anti-apoptotic environment, and alter immune system functions. Accordingly, the production of an immunosuppressant milieu may facilitate tumor escape from immune surveillance and induce chemo-resistance. In this review we will focus on BCR-ABL-independent mechanisms, analyzing especially those with a potential clinical impact in the management of CML patients.

## Introduction

Chronic myeloid leukemia (CML) is a clonal myeloproliferative disorder of pluripotent hematopoietic stem cells (HSCs). CML is hallmarked by a single acquired genetic abnormality, the Philadelphia chromosome (Ph), resulting from a reciprocal translocation between the Abelson leukemia virus (ABL) oncogene from long arm of chromosome 9, and the breakpoint cluster region (BCR) from long arm of chromosome 22. This translocation results in the fusion of the ABL gene to the BCR gene on chromosome 22, and the subsequent generation of the chimeric BCR-ABL1 gene, the molecular counterpart of the abnormal translocation. The BCR-ABL1 protein is a constitutively activated tyrosine kinase, which causes anomalous activation of intracellular signal transduction pathways, leading to an unstable genome, abnormal cellular proliferation, and amplification of CML clones (1). This translates in a differentiation arrest, with an accumulation of immature HSCs into the bone marrow (BM) and the peripheral blood (PB) (1).

Since Imatinib has first been approved by FDA as a tyrosine kinase inhibitor (TKI) in 2001, four additional TKIs, namely dasatinib, nilotinib, bosutinib, and ponatinib, have been granted approval and have become important in the of management CML patients during their path to cure.

**Figure 1:**
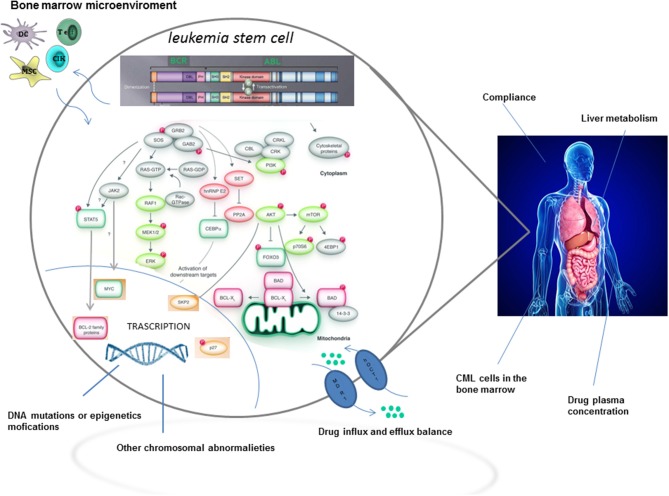
TKI resistance: mechanisms and pathways.

Although TKIs have dramatically changed the treatment of CML by inducing long term overall survival rates higher than 90%, approximately one quarter of patients develop TKI resistance at some point during therapy (2). The most relevant mechanisms and pathways of TKI resistance are detailed in Figure 1. Briefly, resistance to targeted therapies may either be primary or acquired (2). Primary resistance is defined as the lack of response to treatment, whereas acquired resistance is defined as the disease progresses after an initial response to therapy. Notably, acquired resistance develops during treatment, implying that the tumor has developed a mechanism to evade the continuous blockage of the target (3). Point mutations in the BCR-ABL kinase domain are the most frequent mechanisms of acquired resistance development (4), with disease progression and exposure to multiple TKIs being the major players in influencing its frequency.

On the contrary, the overexpression of ATP-binding cassette (ABC) transporters, such as P-glycoprotein (ABCB1) and breast cancer resistance protein (ABCG2), have been implicated as potential mechanisms of primary resistance, as ABC transporters are involved in the regulation of intracellular drug accumulation. Interestingly, higher doses of TKI, specifically imatinib, dasatinib, and nilotinib are able to overcome ABC-related resistance *in vitro* (5), suggesting a possible involvement of ABC-transporters in the development of resistance.

However, it is now becoming clear, from novel biological evidences, that curative approaches in CML patients resistant to TKIs, have not only to consider BCR-ABL-dependent, but also BCR-ABL-independent mechanisms of resistance (2), with a special focus on leukemia stem cells (LSCs). In fact, LSCs may persist in CML patients independently from BCR-ABL1 kinase activation. Moreover, the activation of pathways intrinsic and extrinsic to LSCs may be mediated through upstream and downstream signaling. In this regard, the interaction, within the hematopoietic *niche*, between LSCs and cells from the microenvironment might favor the development of resistance (2). Last but not least, a relatively new concept considers molecular minimal residual disease (MRD) during TKI treatment as the consequence of LSCs persistence. Accordingly, MRD positivity could perhaps be implicated, in the long run, in the development of a TKI resistance.

In this review, we will focus on BCR-ABL-independent mechanisms that have a potential clinical impact for the therapeutically management of CML patients. Of note, BCR-ABL independent mechanisms of resistance to TKI are likely different from those involving resistance or failure after allogeneic-transplant, which are mainly related to dysregulation of the immune system. The discussion of the latter two is not within the scope of the present paper.

## BCR-ABL Independent Mechanisms of Resistance

Treatment with TKIs has revolutionized CML treatment, by inducing high rates of molecular responses in chronic phase. However, a significant proportion of patients still develop resistance, mainly due to the inability of TKIs to kill LSCs, which are responsible for propagating and regenerating CML (6). In essence, LSCs and HSCs share various molecules involved in the maintenance of stemness, such as transcription factors, signal transduction factors, regulators of cell cycle, metabolism, autophagy, and niche-associated factors. However, LSCs and HSCs also have different biological properties influencing their relationship with all the actors involved in self-renewal, providing potential therapeutic opportunities (7).

### Leukemia Stem Cells

#### Heterogeneity of LSCs

In most CML patients treated with TKIs, LSCs are not entirely killed, thus acting as a reservoir of tumor cells that can eventually lead to relapse upon therapy discontinuation, even in patients with undetectable disease. Moreover, CML-LSCs are selectively resistant to TKIs, at least in part, thus reflecting a certain degree of intratumoral heterogeneity, with different responses to treatment in distinct tumor subpopulations. However, up to now, it has been very difficult to characterize CML-LSCs during the remission period, due to their low frequency in the bone marrow, and to the inability to distinguish them from their normal counterpart using standard approaches (8, 9).

Thus, it is still not clear whether TKI-resistant CML-LSCs result from the persistence of a pre-existing therapy-resistant CML-LSCs subset, or if they represent a resistant population which develops as a result of the therapeutic selection process (9).

Some answers to these questions came from single cell genomics, which was able to show CML stem cell heterogeneity and changes resulting from TKI therapy. In a study from the Lund University, 22 patients with chronic phase (CP) CML both at diagnosis and after 3 months of TKI treatment were analyzed (10). The authors combined large-scale single-cell gene-expression analysis with cell surface marker screens, and tried to find a correlation between these techniques. Interestingly, they were able to demonstrate changes in the composition and phenotype of CML-LSC compartment, upon TKI treatment. CML-LSCs have an aberrant expression of cell surface molecules such as CD33, CD123, IL1RAP, CD26, and CD25. These markers can be used, in clinical practice, to distinguish CML-LSCs from normal HSCs. At diagnosis, CD25, CD26, and ILRAP were commonly expressed on CML LSCs, but were then downregulated in some subpopulations in response to TKI treatment (10). Bone marrow Ph^+^ CML CD34^+^/CD38^−^ LSCs were found to specifically co-express CD26 (dipeptidylpeptidase-IV). Regarding the latter, a recent paper showed the persistence of CD26^+^ LSCs in peripheral blood of CML patients in molecular response, receiving TKI treatment or after TKI discontinuation (11). This study confirmed, in an elegant manner, that TKIs are not able to kill LSCs, which usually persist during TKI therapy, thus representing a reservoir of tumor cells potentially responsible for clonal evolution and progression to accelerated or blastic phase.

CML-LSCs were also analyzed by combining high sensitivity mutation detection with whole-transcriptome analysis of the same single cell (9). This technique allowed the identification of novel pathways of potential interest as therapeutic targets. Among others, the marked overexpression of inflammation-associated genes, including those involved in the transforming growth factor (TGF)-beta and tumor necrosis factor (TNF)-alfa pathways were the most interesting as potential therapeutic targets. Furthermore, it also identified an informative molecular signature associated with non-leukemic hematopoietic stem/progenitor cells (HSPCs) (BCR-ABL negative cells from CML patients) at diagnosis, which was shown to be predictive for subsequent therapy resistance. Non-leukemic HSPCs from CP-CML patients showed dysregulation of inflammatory TNF-alpha and TNF-beta pathways, associated with increased SC quiescence and a possible disruption of the CML microenvironment. Interestingly, this finding was also supported by a CML mouse models. Accordingly, elevated serum level of TNF-alpha and TNF-beta might correlate with poor treatment response in CML, indicating that targeting inflammatory pathways might have a therapeutic value (9).

#### Quiescence

CML-LSCs cell-cycle quiescence, intended as their long-term resting capacity, is probably one of the most critical mechanisms of LSC-mediated resistance to TKI (12). It is well-known that: (1) BCR-ABL activity is not necessary for LSCs survival; and (2) TKIs are not able to kill quiescent LSCs. Accordingly, LSCs persistence must be regulated by other aberrant pathways (13).

Recently, it has been identified that a highly quiescent subpopulation of CML-LCSs are able to persist during prolonged period of TKI therapy (8). Interestingly, this subpopulation is molecularly distinct from normal HSCs, due to the overexpression of a number of putative therapeutic targets (TGF-beta, TNF-alfa, Jak-STAT, CTNNB1, and NFKB1A) that might allow for selective targeting of these highly resistant CML-LSCs (8).

A few years ago, Neviani el al. identified protein phosphatase 2A (PP2A), a tumor suppressor able to decrease survival and self-renewal capacity of quiescent CML-LSCs, but not of normal quiescent HSCs (13). Interestingly, PP2A exerts its action on CML-LSCs through the inhibition of JAK2 and β-catenin, which is completely BCR-ABL independent. BCR-ABL1 expression in quiescent CML-LSCs, but not its activity, allows for the recruitment and the activation of other oncogenes, such as JAK2, and it enhances β-catenin activity. Furthermore, BCR-ABL1 expression induces SET-mediated inactivation of PP2A, whose lost activity in CML can be reestablished by PP2A activating drugs FTY720 (14–17). In an elegant experiment, PP2A activator FTY720 was shown to be ineffective for normal HSCs, but strikingly damaged survival and self-renewal of quiescent CML-LSCs. Unexpectedly, the major determinant for these effects on quiescent CML-LSCs was the PP2A-induced inactivation of JAK2 and β-catenin, and not BCR-ABL1 inactivation.

Another interesting point to discuss is the relationship between autophagy and resistance. Autophagy is a conserved catabolic process responsible, between others, for protein degradation and antigen presentation. In CML, TKIs treatment is responsible for the development of autophagy, which favors LSCs survival and TKI-resistance (18). Accordingly, the selective inhibition of autophagy might revert TKI resistance and/or target CML-LSCs, causing their death (19–21). Baquero et al. have investigated, in a xenotransplantation model of human CML, the effect of autophagy inhibition with Lys05, a highly potent lysosomotropic agent, and PIK-III, a selective inhibitor of the vacuolar protein sorting 34 (VPS34), on the survival and function of LSCs (18). This paper reported three interesting findings: (1) basal autophagy is higher in CML-LSCs with respect to normal HSCs; (2) autophagy inhibition with Lys05 significantly reduce LSCs quiescence and promotes myeloid cell expansion; (3) Lys05 and PIK-III combined treatment significantly decrease the number of primary CML-LSCs, and is able to kill xenografted LSCs when used together with TKIs (18). These data indicate a possible role for combining TKIs and second-generation autophagy inhibitors, paving the way for considering them as a potential option in the treatment armamentarium for CML patients with MRD (18), as the persistence of LSCs after TKI treatment has been associated with disease relapse.

#### Signaling Pathways and Transcription Factors

An important limitation of imatinib, the first-line treatment for the vast majority of worldwide CML patients, is that survival of CML-LSCs is not dependent from BCR-ABL activity. Accordingly, CML-LSCs are not eliminated during imatinib therapy, meaning that they use survival signals different from BCR-ABL to survive and resist during imatinib treatment (22, 23).

By using a large-scale RNA interference (RNAi) screen to identify genes involved in imatinib responsiveness, Ma et al. discovered an increase activity in RAF/MEK/ERK signaling pathway responsible for BCR-ABL-independent imatinib resistance through CML-LSCs (24). In BCR-ABL independent imatinib resistant cells, the increased expression of the protein kinase C (PKC) family member PKCη sustains the RAF/MEK/ERK signaling, leading to CML cell proliferation and inhibition of apoptosis (24). In this condition of BCR-ABL independent imatinib resistance, treatment with imatinib sustains RAF/MEK/ERK signaling, giving additional proliferative advance to CML-LSCs (24). Indeed, to overcome BCR-ABL independent resistance to Imatinib, it might be necessary to simultaneously inhibit both BCR-ABL and RAF/MEK/ERK signaling (24). With this in mind, Imatinib and Trametinib, a MEK-inhibitor, were recently combined in a mice model, showing successful killing of CML-LSCs (24), and providing the proof of principle to test this combination in humans also.

Gerber et al. (25), using exon microarrays, performed genome-wide transcriptome analysis of highly purified CML-LSCs and normal HSCs, to better characterize gene-expression profile of CML-LSCs, and find possible therapeutic marks specific for this subpopulation. Ninety-seven differentially expressed genes were found in CML-LSCs vs. normal HSCs. A lot of genes crucial for cell metabolism, cell proliferation, cell surface, self-renewal, pro-differentiation, and inflammation were either upregulated or downregulated in CML-LSCs vs. normal HSCs (25). Indeed, the differences in gene-expression may explain, at least in part, the biological characteristics of CML-LSCs, comprising their increased proliferation capacity, altered oxidative metabolism and resistance to apoptosis (25). Interestingly, some of the over-expressed genes in CML-LSCs encode cell surface proteins, such as IL2Rα (CD25), DPP4 (CD26), PTPRD, CACNA1D, IL1RAP, SLC4A4, and KCNK5. The location of these proteins on the cell surface make them possible good candidates for targeting with immune-based strategies. In this regard, at present 3 cell surface molecules are probably more interesting than the others. The first target is DDP4 (CD26), which is aberrantly expressed by CML-LSCs. DPP4 targets CXCL12 with its peptidase cleavage activity, and its upregulation may favor a dysregulated growth and survival of CML-LSCs through the escape of the homing/niche interactions imposed by the CXCL12/CXCR4 chemokine-receptor system (26, 27). The selective inhibition of DPP4, proven to be successful for the treatment of diabetes, may thus also be explored in CML, with the aim of selectively killing CML-LSCs. The second one is IL2Rα (CD25), with several blocking antibodies currently in development, especially for neurological disorders such as multiple sclerosis (28). Last but not least, IL-1 receptor antagonists blocking IL1RAP, already FDA-approved for the treatment of several inflammatory disorders, could also potentially be used to target CML-LSCs (29).

### Epigenetics Events

Cancers are, at least in part, epigenetically-driven diseases. It is well-established that epigenetic dysregulation is as important as genetic abnormalities for tumor birth, maintenance and progression. Growing evidence indicates that epigenetic dysregulation have also a causal role in TKI resistance, leading to leukemic clone escape and disease propagation (30).

Epigenetic change is initiated and sustained by at least three different systems, including histones and histone modifications, DNA methylation, and non-coding RNAs. Histone acetyl- or methyl- transferases or histone deacetylase or demethylases are enzymes able to add or remove variations, respectively, at specific amino acid residues or CpG islands in DNA, whereas anti sense transcripts and miRNAs can regulate mRNA levels and protein translation (31–33). Together, they can directly regulate transcription by acting on DNA damage/repair and DNA replication, modulate RNA levels and stability post-transcriptionally, or have an impact on protein translation or post-translational protein modifications (34). An association between CML progression and resistance to TKI treatment and of CpGs islands was recently demonstrated (35, 36). Moreover, an higher methylation of transcription factor AP-2 alpha (TFAP2A) and early B-cell factor 2 (EBP2) was found in patients with blastic phase with respect chronic phase, and autophagy related 16-like 1 (ATG16L1) was methylated in 69% of CML patients. Finally, the probability of achieving a major molecular response (MMR) at 12 or 18 months was lower in methylated with respect to unmethylated cases at baseline (35, 36).

In CML, several histone marking systems are dysregulated, affecting several survival pathways for leukemic cells. Among others, SIRT1 deacetylase is a multifunctional protein regulating the acetylation of several transcription factors, including p53 (37), Ku70, and FoxOs (38). SIRT1 expression is higher in human CML CD34^+^ cells than in normal CD34^+^ cells (39, 40). It has recently been demonstrated that SIRT1 deacetylase on the one hand favors acquisition of genetic mutations in CML cells leading to TKI resistance, an on the other hand is critical for keeping CML-LSCs alive (39, 40). The inhibition of SIRT1 inhibits LSCs growth *in vitro* and *in vivo* and promotes CML-LSCs apoptosis in both chronic and blastic phase (40). Interestingly, SIRT1 inhibition had no significant effect on normal CD34^+^ cells proliferation and apoptosis, thus introducing SIRT1 inhibition as a possible new frontier for eliminating CML-LSCs.

Other epigenetic pathways frequently deregulated in both solid and hematological cancers are the polycomb repressive complex 1 (PRC1) or complex 2 (PRC2) (41). Regarding PRC1, its oncogene member BMI1, a downstream target of the BCR–ABL1 tyrosine kinase, was identified as a possible prognostic marker in CML (42, 43). The expression of high levels of BMI1, typically found in patients with advanced phase CML, were linked to a poorer outcome (44). On the contrary, a high expression of 2 other polycomb group multiprotein PRC1-like complex, chromobox protein homolog 6 and 7 (CBX6 and CBX7) was associated with a favorable outcome (44). Finally, time to obtain cytogenetic response and event free survival were also negatively conditioned by the expression of some PRC1 genes (43). Concerning the epigenetic “writer” complex PRC2, it is central for the definition of stem-cell identity, as it predominantly modulates gene repression using trimethylation of lysine 27 on histone H3 (H3K27me3) (45).

In both solid and hematological malignancies, aberrant H3K27me3 and EZH2 activity have been implicated poor prognosis or rapid progression. PRC2 dysregulation in primary CML cells, cell lines or murine models has been demonstrated. Furthermore, PRC2 expression levels may be altered in response to TKI (45, 46) or progression to blastic phase (47). Conversely to normal HSPC, CML-LSC showed an increased dependency of pro-apoptotic genes on PRC2-EZH2 repression. Treatment of primary CML cells with either EZH2 inhibitors or TKIs alone significantly upregulated H3K27me3 targets, and combined treatment with TKIs and EZH2 inhibitors significantly killed CML-LSCs, both *in vitro* and in bone marrow murine xenografts (34).

The last epigenetic pathway involved in CML resistance are miRNAs, a family of small, non-coding RNAs consisting of 19–22 nucleotides, which affect gene expression through binding to 3′-UTR within target mRNAs. Aberrant miRNAs exhibit a global down-regulation in cancers (48), suggesting miRNAs are tumor suppressors at overall level. Recently, a miRNA microarray study comparing the miRNAs expression in K562 cell line with healthy controls showed a high number of miRNAs down-regulated in K562 (49), opening the possibility to future therapies in CML patients.

Additionally, the degree of down-regulation of several miRNAs, evaluated in drug-naïve chronic phase patients, can be used to distinguish between imatinib responders and non-responders (50–53). These include miR-29 cluster, miR-23a, and miR-451 (50). Interestingly, in a subset of patients responding to imatinib, it has been found that there is an inverse relationship between miR-451 and BCR-ABL1 expression (51–53), probably because miR-451 directly target BCR/ABL1. However, it is too early to directly correlate miRNA levels with response to TKIs. Further studies are warranted to clarify if different miRNA levels in diagnostic samples will be predictive of a different response to different TKIs, or may be predictive for a better or a worst prognosis (34).

Finally, miRNA might have a role in tumor-endothelial crosstalk, within the bone marrow microenvironment, through their exosomal transfer between cell types (42, 54). In CML, BCR-ABL1 suppresses CXCR4-mediated signaling, thus altering the interactions between leukemic cells and the bone marrow stroma, which are defective. In an elegant experimental model, Taverna and coworkers co-cultured CML cell lines with endothelial cells in order to demonstrate that miR-126 secreted from CML cells can be transported to endothelial cells in order to affect their phenotype (54). Their work suggests that CXCL12/CXCR4 signaling may be inhibited *in vivo* through down-regulation of CXCL12 in the stroma by exosomal shuttle of miR-126 from CML cells to stroma (42, 54).

### Microenvironment

It is now clear that a large number of LSCs residing in the bone marrow niche are dormant and resistant to traditional chemotherapies. Surrounding stromal cells may influence LSCs fate by promoting cell cycle arrest through specific signals, thus allowing their persistence even during TKI treatment (55). The maintenance and regulation of stem cells and their progeny, together with long term hematopoiesis are sustained, within the bone marrow microenvironment, by paracrine- and autocrine-derived growth factors and cytokines.

#### Mesenchymal Stromal Cell

Mesenchymal stromal cells (MSCs) are pivotal contributors in the set up and maintenance of the HSC niche and in the development and differentiation of the lympho-hematopoietic system (56). Moreover, MSCs have a distinctive immunomodulatory capacity, which affects the function of immune cells, *in vitro* and *in vivo* (57). Bone marrow MSCs from CML patients do not belong to the Ph1-positive clone. However, the role of BM stromal cells, including MSCs, recently gained attention, as they were considered critical contributors to leukemogenesis and to the protection of CML LSCs from the effects of TKIs.

Recently, MSCs from CML patients were isolated at diagnosis and at achievement of deep molecular response (58). Gene expression profiling of MSCs from CML patients was then compared to MSCs from healthy individuals. Interestingly, MSCs from CML patients exhibit a gene profiling pattern distinct from normal MSCs. In particular, six genes (BMP1, FOXO3, MET, MITF, NANOG, and PDPN) were over-expressed in CML MSCs, persisting in patients with deep molecular response (58). The authors concluded that CML-MSCs show an abnormal gene expression pattern persisting in patients with deep molecular response. Accordingly, it is possible that the abnormal gene expression pattern of MSCs is established during development of CML within the HSC *niche*.

MSCs also have an important role in maintaining normal HSCs and LSCs within the *niche*; recent evidences suggest that CXCL12-expressing MSCs are involved in HCSs and CML LSCs regulation (59). CXCL12 deletion from MSCs expand LSCs, reduces LSCs co-localization, enhance LSCs cycling (59). As a consequence, LSCs undergo increased self-renewing divisions, related to enhanced EZH2 activity. CXCL12 deletion from MSCs, moreover, increase LSC elimination by TKI treatment, thus favoring the eradication of CML LSCs and reducing the probability of CML persistence and recurrence. In brief, CXCL12 MSCs, but not other CXCL12-expressing BM microenvironment cell populations, allow the persistence of quiescent, TKI-resistant LSCs within BM *niches* (59).

#### Soluble Factors

It has recently been investigated how a bone marrow microenvironment can mediate resistance to TKIs. To evaluate the role of the bone marrow microenvironment on imatinib mesylate sensitivity, Bewry et al. used an *in vitro* bone marrow stroma model in order to establish the role of soluble factors in contributing to imatinib resistance (60). K562 cells were cultured in a stroma-derived conditioned medium, and this was sufficient to favor the development of resistance to TKIs by reducing apoptosis induced by imatinib, nilotinib, and dasatinib (60). Moreover, K562 clonogenic potential was higher in conditioned medium with respect to control medium (60). Finally, soluble factors produced by HS-5 cells were able to increase Stat3 levels in K562, whose increased activation has been associated with malignant transformation of several human cancers and drug-resistant tumors (61, 62). Moreover, a stroma-derived conditioned medium was responsible for the increase of K562 cells survival after IM treatment. Furthermore, increased pStat3 levels in K562 cells correlated with increased expression of Stat3-regulated genes Bcl-xl, Mcl-1 and patient's survival after imatinib treatment. When Stat3 levels were reduced using small interfering RNA, apoptosis of K562 induced by imatinib was restored, even when cells were cultured in conditioned medium. In summary, soluble factors secreted by stromal cell activate Stat3 in CML cells, thus causing resistance to TKIs and contributing to the inability of TKIs to eradicate MRD (60). Remarkably, in a previous work, the same group reported that adhesion to fibronectin was sufficient to protect K562 cells from imatinib-induced cell death (63). Accordingly, a leukemic bone marrow microenvironment is able to induce resistance either through the secretion of soluble factor by stromal cells, either through cell-to-cell contact.

#### Immune System and Response to Treatment

There is emerging evidence that the immune system plays a major role also in CML, not only for disease development and progression, but also for prognosis and response to treatment (64). Recently, treatment-free remission for patients in deep molecular response under TKI treatment has emerged as one of the major goals, with the intention of reducing toxicity in those patients without detectable disease. Even if it is difficult to demonstrate the direct relationship between immune system status and response to treatment, studies on TKIs discontinuation have attributed the lack of overt relapse in such patients to immunological control of CML (64, 65). In the initial phases of the disease, the accumulation of immature myeloid cells (myeloid-derived suppressor cells, MDSCs), which originate from the malignant BCR-ABL1 clone, leads to suppression of the innate and adaptive immune system, leading to CML development. In support, quantitative and functional defects of NK cells and reduced cytotoxic T lymphocyte function have been reported in CP-CML patients at diagnosis (66).

Moreover, there are some evidences supporting the correlation between immune system, checkpoint inhibitors, microenvironment and long-term molecular response, that warrant further evaluations.

First of all, the number of MDSC in patients with a deep molecular response reduced following highly efficacious TKI therapy (64, 66–68). Furthermore, a deep molecular response seems to correlate with increased NK-cell and CD8^+^ T-cell counts in the peripheral blood of CML patients (67, 68). Last but not least, levels of checkpoint receptors such as PD1, CTLA4, and TIM3 seems to be higher in CD4^+^ and CD8^+^ T cells of CML patients with respect to control. In addition, PD1 expression is elevated in the bone marrow T-cells compared to paired peripheral blood samples, and it decreased in CD8^+^ T cells during TKI treatment (67, 68). In other words, enhanced net effector immune responses and decreased PD-1 and immune suppressors may promote sustained deep molecular response in CML (67, 68).

Among the regulatory signals, the axis CXCL12/CXCR4, an important regulator of normal hematopoiesis within the bone marrow niche, seems to be involved also in CML. As previously mentioned, even if the exact mechanism is still unclear, CML cells may influence the expression of functional CXCL12 within the tumor microenvironment, thus favoring their egress from bone marrow to peripheral blood (67). Other players potentially responsible for the reduction of CXCL12 functional expression are cytokines and chemokines produced by stromal cells, including IL-1 alpha, IL-1 beta, IL-6, G-CSF, TNF-, CCL3, and CCL4. Among these, only G-CSF *in vitro* decreased CXCL12 expression in bone marrow stromal cells, while anti-G-CSF antibody treatment increased CXCL12 expression and CML-LSC numbers in bone marrow, and reciprocally decreased CML LSC numbers in the spleen (68).

Finally, the immune cell contexture in the BM microenvironment could have an impact in response to TKI in CML patients (69). In a recent paper (69), BM of 56 CML patients at diagnosis and 14 healthy controls were studied by immunohistochemistry and automated image analysis. Interestingly, T-cell exhaustion status was also studied at diagnosis and after 1, 3, and 6 months in both BM and peripheral blood. Briefly, a severe myeloid and lymphoid cell-mediated immunosuppression was found at diagnosis in CML BM, which was partly restored with successful TKI treatment (69). Moreover, higher levels of PD-1, TIM3, and CTLA4 were found in CD4^+^ and CD8^+^ cells of CML patients with respect to healthy controls. Interestingly, the levels of immune checkpoint decreased also during successful TKI therapy, thus indicating a clear relationship between immune system status and response to TKI (69). Moreover, the authors developed a novel risk stratification model predicting the probability of achieving a deep molecular response (MR 4.0). Briefly, low CD4^+^ T-cell proportion, high proportion of PD1^+^TIM3^−^CD8^+^ T cells, and high PB neutrophil count were most predictive of lower MR4.0 likelihood (69). Furthermore, the combination of low CD4^+^ T-cell proportion and high PB neutrophil counts was tested in a validation cohort (*n* = 52) analyzed with flow cytometry. Interestingly, these two variables were still also predictive of a lower MR4.0 likelihood in the validation cohort. This observation, if confirmed in larger series, may be the proof of principle that immune biomarkers may be used to predict molecular response in CML patient treated with TKI. Consequently, this study could map the road to further test immunomodulatory drugs in CML treatment.

## Strategies to Fight BCR-ABL Independent Resistance

It is now quite clear that if acquired resistance is mainly due to point mutation, MRD persistence is the result of BCR-ABL independent drug resistance. As stated earlier, CML-LSCs residing in the bone marrow are protected from TKI-induced killing, even at high doses (70). Accordingly, if the final objective is to eliminate CML-LSCs, and this is probably the prerogative only for few patients, it becomes evident that TKIs have to be combined with other drugs. A list of the most relevant clinical trials dealing with TKI resistance in CML are listed in Table 1.

**Table 1:** Clinical trials for TKI resistance CML patients.

**Drug**	**Title**	**Target**	**Rationale**	**Patient population**	**Primary endpoint**
Ruxolitinib in combination with nilotinib	Phase I/II study of nilotinib/ruxolitinb therapy for TKI resistant Ph-leukemia	JAK/STAT5	To assess if the combination approach of nilotinib with ruxolitinib could block alternative pathway besides BCR-ABL kinase inhibition in Ph positive leukemia, esp against JAK2-STAT5 pathway	TKI resistant CML or ALL patient	Phase I: MTD of ruxolitinib with fixed dose of nilotinib Phase II: major cytogenetic response
Smoothened (SMO) inhibitor BMS-833923 plus dasatinib	Dasatinib combination therapy with the smoothened (SMO) inhibitor BMS-833923 in chronic myeloid leukemia (CML)	SMO	The purpose of the study is to determine the safety and tolerability of the combination of BMS-833923 plus dasatinib in CML patients	Resistance or suboptimal response to imatinib, dasatinib, or nilotinib and no known T315I/A Abl-kinase mutation CML or Ph^+^ ALL patients	Recommended Phase 2 dose (RP2D) of BMS-833923 Plus dasatinib in chronic myeloid leukemia-chronic phase [time frame: day 1 to week 80, with observation for DLT in weeks 5–8]
Ponatinib	Ponatinib in participants with resistant chronic phase chronic myeloid leukemia (CP-CML) to characterize the efficacy and safety of a range of doses	BCR-ABL	To characterize the efficacy of ponatinib administered in three starting doses [45 milligram (mg), 30 mg, and 15 mg daily] in CP-CML patients who are resistant to prior TKI therapy or have T315I mutation	CP-CML who are resistant to prior TKI therapy or have T315I mutation	Percentage of participants with ≤ 1% BCR-ABL1IS at month 12
Ponatinib	A study in patients with chronic leukemia, where previous therapy failed, and who will be treated with ponatinib as second line therapy (PONS)	BCR-ABL	The aim of the study is to evaluate the safety and efficacy of ponatinib as a II line treatment in patients failing or not tolerating I line therapy with any other approved TKIs	CP-CML patients who were treated with TKI in a previous therapy but which has not been effective	Major molecular response (MMR) of treatment
Dasatinib	Treating patients with CML in chronic phase (CP) with dasatinib	BCR-ABL	This non-interventional study is designed to collect real-life data on CML-treatment with dasatinib in clinical routine with respect to first and second line treatment and/or switch setting (within 1st line or from 1st line TKI to 2nd line dasatinib)	Patients with newly diagnosed CP-CML and CML patients in chronic phase resistant or intolerant to prior therapies, including imatinib. Any line treatment of chronic CML	Distribution of molecular remission status at study entry and after 12 months

Using a combination of Ruxolitinib and Nilotinib, Gallipoli and colleague detected an increased apoptotic rate in CML cell lines and a decrease of engraftment in CML murine models (71). This preclinical study proposed JAK2-TYK2-STAT3 as a crucial pathway to target in order to overcome bone marrow microenvironment-mediated drug resistance, potentially leading to MRD eradication. Consequently, in a phase I study, CML patients were treated with Nilotinib plus Ruxolitinb to determine the maximum-tolerated dose of ruxolitinib and establish a toxicity profile. This combination was safe and well-tolerated, without significant worsening in patient-experienced fatigue (72).

In order to target the epigenome together with BCR-ABL, the combination of TKI and epigenetic drugs, such as decitabine or azacytidine, was recently explored. Few preliminary clinical trials showed that treatment with hypomethylating agents is able induce responses in CML patients, even in advanced stages and despite imatinib-refractoriness (73). However, responses to this combo are usually not durable, and might be considered only as a possible bridge to transplant in patients not responding to TKIs.

Other interesting drugs are the novel, orally administered EZH2 inhibitors, currently being tested in clinical trials. These drugs have low toxicity, produce significant reductions of H3K27me3 levels, and are capable of achieving promising objective responses across a wide range of tumor types. If these data are be confirmed, this should be a promising new avenue for epigenetic therapy in CML, especially considering the importance of PRC1 and PRC2 in CML (45).

Recently, peroxisome proliferator activated receptor (PPAR)-c agonists, including the drug pioglitazone, have been reported to be able to target CLM LSCs pool in biological assays. Anecdotally, three clinical CML cases were treated for associated type 2 diabetes or off-label (74). Fifteen PPAR-c agonists are currently used as antidiabetic drugs that are not hypoglycemogenic in healthy individuals. Quiescent CML-LSCs are resistant to TKIs, but pioglitazone pulls them out quiescence, and consequently sensitizes them to imatinib. Pioglitazone in combination with imatinib was tested in a phase II trial (ACTIM study) in CML patients not achieving MR4.5 with imatinib alone. The results of the ACTIM study suggested that the pioglitazone and imatinib combination increases the proportion of CML patients achieving MR 4.5. These results provide a proof of concept needing confirmation in a controlled, phase III, randomized clinical trial (74).

Finally, as epigenetic modifications are now more and more recognized as critical mechanisms of CML pathogenesis, progression, and TKI resistance, new treatments targeting different checkpoints of the epigenome very frequently originate from bench to bedside. Recently, p53 stabilization combined with BET inhibitor-mediated chromatin disruption was able to efficiently kill CD34^+^ cells from CML patients, highly enriched for CML-LSCs (75). Furthermore, BET inhibitors can downregulate PD-L1 expression on solid tumor cells (76, 77), thus facilitating the activation of immune checkpoints and long-term LSC clearance, in a similar manner to the suppression of PD1 on T cells in CML patients with TKI-induced deep molecular response (66). Unfortunately, responses to epigenetic drugs seem to be brief in the clinical setting, due to early development of resistance. However, resistance to BET inhibitor seems to be driven by preexisting highly adaptive clones with a high degree of transcriptional plasticity, which can revert BET sensitivity when treatment is discontinued. In conclusion, epigenetic factors will surely continue to play a role in CML treatment and prognosis. Nonetheless, the elucidation of critical pathways in cancer initiation, maintenance, and successful elimination of LSC will continue to benefit from CML research.

## Conclusions

The vast majority of patients with CML control the disease through continuous TKI treatment. However, only a small minority of these patients are able to retain remission after TKI discontinuation. In other words, if disease control is likely to happen in all but few patients, the cure rate is still low. The persistence of a MRD in most CML patients, sometimes undetectable by standard techniques, is due to a pool of TKI-persistent and BCR–ABL1 kinase–independent LSCs. LSCs in CML are defined as Ph^+^ CD34^+^CD38^−^ primitive progenitor cells with a higher capacity to engraft in immunocompromised mice with respect to normal CD34^+^ cells, stem-cell properties such as quiescence and self-renewal, genomic instability, and resistant to apoptosis. Furthermore, in the bone marrow of CML patients, LSCs take the advantage from an immunosuppressive milieu maintained from the tumor microenvironment, which is permissive and leukemia-favorable. These mechanisms of resistance, BCR-ABL independent, are responsible for TKI failure, and are probably involved in a large number of cases of secondary resistance and in disease recurrence after TKI discontinuation. As a consequence, novel therapeutic approaches targeting LSCs are currently being tested and developed, in order to address an important unmet clinical need for a significant proportion of patients with unsatisfactory results with TKIs. In conclusion, even if TKIs have dramatically changed CML history, we are still far from a curative approach able to target LSCs and, thus, cure a large number of CML patients. The combination of new drugs targeting the microenvironment, or the epigenome or metabolic pathways responsible for the persistence of CML LSCs will probably increase the cure rate, by targeting BCR-ABL independent mechanisms of resistance, responsible for TKI failure.

## Author Contributions

FL and AI wrote the manuscript. GV, SG, and AC extensively commented on the manuscript.

### Conflict of Interest

The authors declare that the research was conducted in the absence of any commercial or financial relationships that could be construed as a potential conflict of interest.
